# Prevalence of parental stress and associated factors among parents of children with pediatric psychiatric disorders: a cross-sectional observational study

**DOI:** 10.1192/j.eurpsy.2026.11114

**Published:** 2026-07-31

**Authors:** S. Essadki, F. Wahoud, W. Hajjaji, A. Zarouali, R. Lamsyah, S. Mahfoudi, A. Ouamar, A. Amara, I. Sehb, H. Elmsellem, A. El Alaiki, I. Douelfiqar, A. Yacoubi, M. A. Lafraxo

**Affiliations:** 1 Higher Institute of Nursing Professions and Health Techniques; 2 Laboratory of Maternal, Infant, and Mental Health(LSMIM), Faculty of Medicine and Pharmacy, Oujda; 3 Higher Institute of Nursing Professions and HealthTechniques, Fez; 4 The Hospital of Mental Health and Psychiatric Diseases of the CHU Mohmamed-VI, Oujda; 5 Faculty of Science, Ibn Tofail University, Kenitra; 6 Laboratory of Epidemiology, Clinical Research, and Public Health, Faculty of Medicine and Pharmacy, Mohammed First University, Oujda; 7 Laboratory of Biology and Health, Faculty of Science, Ibn Tofail University, Kenitra, Morocco

## Abstract

**Introduction:**

Parental stress is a phenomenon frequently observed among parents of children with pediatric psychiatric disorders. The complexity of care, the nature of the disorders, and the resulting emotional and economic impacts can exacerbate this stress.

**Objectives:**

The objective of the present study is to assess the level of parental stress among parents of children with pediatric psychiatric disorders and to identify the main factors associated with it.

**Methods:**

This is a cross-sectional observational study conducted at the Hospital of Mental Health and Psychiatric Disorders, affiliated with the Mohammed VI University Hospital Center in Oujda, Morocco. The study included a sample of 85 parents of children aged 3 to 17 years presenting various pediatric psychiatric disorders. Data were collected using a socio-demographic questionnaire and the Parental Stress Index Scale (PSI).

**Results:**

According to the results, the mean parental stress score was 115.41 (±19.32). Among the parents surveyed, 35.3% exhibited a very high level of stress. Statistical analysis identified several factors significantly associated with parental stress, including sex (p< 0.001), number of children (p = 0.034), difficulties with falling sleep (p = 0.046), lack of personal time or time for other family members (p = 0.037), difficulty organizing appointments (p< 0.001), loss or reduction of income related to the child’s illness (p = 0.031), social isolation (p=0.006), feelings of misunderstanding or judgment from close family members (p = 0.020), and feelings of guilt regarding the child’s situation (p = 0.033). These findings indicate that parental stress is influenced by a combination of sociodemographic, emotional, and contextual factors.

**Conclusions:**

The results of this study highlight a particularly high level of parental stress among parents of children with pediatric psychiatric disorders. The main factors associated with this stress include sex, emotional burden, social isolation, and organizational difficulties related to the care of the child. These findings underscore the need to develop targeted support and psychological assistance programs aimed at enhancing parental coping capacities and providing continuous support throughout the child’s care pathway.

**Disclosure of Interest:**

None Declared

